# Effect of basal metabolic rate on lifespan: a sex-specific Mendelian randomization study

**DOI:** 10.1038/s41598-023-34410-6

**Published:** 2023-05-12

**Authors:** Jack C. M. Ng, C. Mary Schooling

**Affiliations:** 1 School of Public Health, Li Ka Shing Faculty of Medicine, The University of Hong Kong, Pokfulam, Hong Kong Special Administrative Region China; 2 Department of Environmental, Occupational, and Geospatial Health Sciences, Graduate School of Public Health and Health Policy, The City University of New York, 55 West 125th St, New York, NY 10027 USA

**Keywords:** Evolutionary theory, Endocrine system and metabolic diseases, Epidemiology, Medical research, Genetics research

## Abstract

Observationally, the association of basal metabolic rate (BMR) with mortality is mixed, although some ageing theories suggest that higher BMR should reduce lifespan. It remains unclear whether a causal association exists. In this one-sample Mendelian randomization study, we aimed to estimate the casual effect of BMR on parental attained age, a proxy for lifespan, using two-sample Mendelian randomization methods. We obtained genetic variants strongly (p-value < 5 × 10^–8^) and independently (r^2^ < 0.001) predicting BMR from the UK Biobank and applied them to a genome-wide association study of parental attained age based on the UK Biobank. We meta-analyzed genetic variant-specific Wald ratios using inverse-variance weighting with multiplicative random effects by sex, supplemented by sensitivity analysis. A total of 178 and 180 genetic variants predicting BMR in men and women were available for father’s and mother’s attained age, respectively. Genetically predicted BMR was inversely associated with father’s and mother’s attained age (years of life lost per unit increase in effect size of genetically predicted BMR, 0.46 and 1.36; 95% confidence interval 0.07–0.85 and 0.89–1.82), with a stronger association in women than men. In conclusion, higher BMR might reduce lifespan. The underlying pathways linking to major causes of death and relevant interventions warrant further investigation.

## Introduction

Metabolism has long been linked to the process of aging and longevity but the evidence from studies of their associations is not always in accordance^1^. Several different theories of aging related to metabolism exist particularly within a life history context, where reproductive strategies and lifespan are seen as shaped by natural selection^2^, such as the “rate-of-living” theory and the “free radical” theory. The “rate-of-living” theory suggests that faster metabolism accelerates aging and therefore reduces lifespan whereas the “free radical” theory suggests that oxidative stress arising from metabolism is toxic and the harm accumulates to damage body systems^3^. Correspondingly, on average, men have a higher metabolic rate^4^ and also have shorter lives than women.

Total daily energy expenditure consists of basal metabolic rate (BMR), thermic effects of food, and energy expenditure from physical activity^5^. BMR reflects the daily energy requirement for maintaining basic bodily functions. It is the major source of energy expenditure^6^ and is an important parameter for estimating daily energy requirements^7^.

Observational studies have suggested that higher BMR predicts mortality in young individuals^8,9^ but lower mortality in old individuals^10^. Somewhat counter-intuitively, associations that change with age at recruitment may indicate causality because each successively older age group are more strongly selected survivors of a harmful exposure^11^. Evidence as to whether changing BMR affects the risk of mortality is lacking. In addition, observational studies are susceptible to residual confounding, when factors affecting both BMR and mortality, such as body composition, exist. A study in mice suggested that the observational association of BMR with lifespan could be confounded by fat mass^12^. Notably, body composition was not adjusted for in some of the human studies assessing the association of BMR with mortality^9,10^. Some forms of physical activity, such as resistance training but not aerobic exercise, have been shown to increase BMR^13^, but cannot be comprehensively assessed and adjusted for adequately, as seen in previous human studies on the same topic^9,10,14^. People with metabolic disorders, such as type 2 diabetes, have a higher BMR than those without^15,16^ and are also at a higher risk of mortality, although a change in BMR could have occurred due to latent metabolic disruption making it look as if BMR caused the disease^17,18^. Aging and progression of some other medical conditions may lower BMR over time^19^ and increase the risk of mortality, which might also obscure the true associations. To date, no randomized controlled trial targeting BMR as a means of increasing lifespan has been conducted.

Mendelian randomization (MR) uses genetic variants (GVs) as instrumental variables to estimate the causal effect of an exposure on an outcome^20^. Since alleles are randomly assorted and are fixed at conception, MR studies have the advantage over conventional observational studies of being less vulnerable to confounding. MR studies of longevity have the advantage of ensuring that exposure allocation and recruitment are aligned, thereby avoiding any time-related biases^21^, such as selection or survival bias.

We conducted a sex-specific MR study to estimate the causal effect of BMR on parental attained age, a proxy of lifespan, because BMR differs by sex^22^. For validation, we also included survival to old age as an additional outcome.

## Results

### Genetic predictors of basal metabolic rate

We obtained 217 and 219 GVs strongly and independently predicting BMR in men and women, respectively, of which 178 and 180 were available for father’s and mother’s attained age (Supplementary Table S1). Correspondingly, these GVs explained 6.5% and 5.6% of the variance in BMR, with a mean F-statistic of 62 (range 30–363) and 61 (range 30–400) (Supplementary Table S2). Supplementary Table S3 shows the GVs predicting BMR that were associated with potential confounders. The study has power of > 80% to detect 0.59 and 0.67 years of life changed per one unit of effect size increase in genetically predicted BMR in men and women, respectively.

### Association of basal metabolic rate with parental attained age

BMR was inversely associated with father’s (years of life lost per unit increase in effect size of genetically predicted BMR, 0.46 [95% confidence interval (CI) 0.07–0.85]) and mother’s attained age (years of life lost, 1.36 [95% CI 0.89–1.82]) using inverse-variance weighting (IVW) with multiplicative random effects, with evidence of stronger associations in women than men (Table 1). Sensitivity analyses gave directionally similar results. MR-Egger intercept tests did not suggest the presence of directional pleiotropy. I^2^_GX_ ranged from 0.77 to 0.83 in all MR-Egger analyses.

**Table 1:** Mendelian randomization of basal metabolic rate (Neale Lab) on father's and mother's attained age (Pilling et al.). CI: confidence interval; GV: genetic variant; IVW: inverse-variance weighting; LL: lower limit; MR: Mendelian randomization; MR-RAPS: Mendelian Randomization Robust Adjusted Profile Score; MR-PRESSO: Mendelian Randomization Pleiotropy Residual Sum and Outlier; SE: standard error; UL: upper limit; WM: weighted median.

Exposure		Outcome		Selection of GVs	No. of GVs	MR method	Beta				Years of life lost				p-value	Cochran's Q		MR-Egger			Outlier detected by MR-PRESSO	Sex difference for years of life lost	
Trait	Sex	Trait	Sex				Estimate	SE	95% CI LL	95% CI UL	Estimate	SE	95% CI LL	95% CI UL		Statistic	p-value	Intercept	Intercept p-value	I^2^_GX_		z statistic	p-value
Men																							
Basal metabolic rate	Men	Father's attained age	Men	All	178	IVW (random)	0.020	0.009	0.003	0.037	0.46	0.20	0.07	0.85	**0.021**	360.839	1.14E−14	–	–	–	–	2.894	**0.004**
				All	178	WM	0.022	0.010	0.002	0.042	0.50	0.24	0.04	0.96	**0.035**	–	–	–	–	–	–	1.946	0.052
				All	178	MR-Egger	0.004	0.023	–0.041	0.049	0.10	0.52	–0.93	1.12	0.855	359.698	1.07E−14	0.001	0.455	0.826	–	1.300	0.194
				All	178	MR-RAPS	0.021	0.006	0.009	0.033	0.47	0.14	0.20	0.75	**0.001**	–	–	–	–	–	–	4.198	**2.70E**–**05**
				All	175	MR–PRESSO (corrected)	0.022	0.008	0.007	0.037	0.50	0.18	0.15	0.85	**0.005**	–	–	–	–	–	rs112875651, rs3184504, rs62396185	2.869	**0.004**
				Exclusion of potentially confounded GVs	162	IVW (random)	0.019	0.009	0.001	0.038	0.44	0.22	0.01	0.86	**0.044**	327.644	1.89E−13	–	–	–	–	2.739	**0.006**
				Exclusion of potentially confounded GVs	162	WM	0.005	0.011	−0.017	0.026	0.11	0.25	−0.39	0.60	0.675	−	−	−	−	−	−	3.330	**0.001**
				Exclusion of potentially confounded GVs	162	MR-Egger	−0.007	0.025	−0.057	0.043	−0.16	0.58	−1.30	0.98	0.783	325.157	2.51E−13	0.001	0.269	0.773	−	1.947	0.052
				Exclusion of potentially confounded GVs	162	MR-RAPS	0.020	0.007	0.007	0.033	0.45	0.15	0.15	0.75	**0.003**	–	–	–	–	–	–	3.930	**8.49E**–**05**
				Exclusion of potentially confounded GVs	159	MR-PRESSO (corrected)	0.021	0.008	0.005	0.038	0.49	0.19	0.11	0.86	**0.012**	–	–	–	–	–	rs112875651, rs3184504, rs62396185	2.877	**0.004**
Women																							
Basal metabolic rate	Women	Mother's attained age	Women	All	180	IVW (random)	0.052	0.009	0.034	0.071	1.36	0.24	0.89	1.82	**1.32E**–**08**	357.236	5.93E–14	–	–	–	–		
				All	180	WM	0.048	0.011	0.025	0.070	1.23	0.30	0.65	1.82	**3.32E**–**05**	–	–	–	–	–	–		
				All	180	MR-Egger	0.046	0.026	–0.004	0.096	1.19	0.66	–0.10	2.49	0.071	357.092	4.31E–14	0.0002	0.789	0.829	–		
				All	180	MR-RAPS	0.054	0.007	0.041	0.067	1.40	0.17	1.07	1.74	**2.22E**–**16**	–	–	–	–	–	–		
				All	177	MR-PRESSO (corrected)	0.051	0.009	0.034	0.068	1.32	0.22	0.88	1.76	**1.77E**–**08**	–	–	–	–	–	rs10774624, rs2102278, rs9843653		
				Exclusion of potentially confounded GVs	161	IVW (random)	0.052	0.010	0.033	0.072	1.35	0.26	0.85	1.86	**1.34E**–**07**	308.741	1.62E–11	–	–	–	–		
				Exclusion of potentially confounded GVs	161	WM	0.056	0.012	0.032	0.081	1.46	0.32	0.84	2.08	**4.40E**–**06**	–	–	–	–	–	–		
				Exclusion of potentially confounded GVs	161	MR-Egger	0.065	0.029	0.008	0.121	1.67	0.74	0.22	3.13	**0.024**	308.331	1.27E–11	–0.0004	0.645	0.771	–		
				Exclusion of potentially confounded GVs	161	MR-RAPS	0.054	0.007	0.040	0.068	1.40	0.19	1.03	1.77	**7.33E**–**14**	–	–	–	–	–	–		
				Exclusion of potentially confounded GVs	159	MR-PRESSO (corrected)	0.053	0.009	0.035	0.072	1.38	0.24	0.90	1.86	**6.95E**–**08**	–	–	–	–	–	rs10774624, rs2102278		

Overall, BMR was inversely associated with parental attained age (Supplementary Table S4).

### Association of basal metabolic rate with survival to 99th and 90th percentile of ages

BMR was not associated with survival to the 99th or 90th percentile of ages using IVW with multiplicative random effects in both men (odds ratio for 99th percentile of age per unit increase in effect size of genetically predicted BMR, 0.92 [95% CI 0.76–1.11]; odds ratio for 90th percentile of age, 0.91 [95% CI 0.80–1.03]) and women (odds ratio for 99th percentile of age, 0.97 [95% CI 0.80–1.18]; odds ratio for 90th percentile of age, 0.94 [95% CI 0.82–1.07]), although the associations were in the direction of higher BMR reducing survival (Table 2). Sensitivity analyses gave similar results. MR-Egger intercept tests did not suggest the presence of directional pleiotropy. I^2^_GX_ ranged from 0.78 to 0.87 in all MR-Egger analyses.

**Table 2:** Mendelian randomization of basal metabolic rate (Neale Lab) on survival to old age (Deelen et al.). CI: confidence interval; GV: genetic variant; IVW: inverse-variance weighting; LL: lower limit; MR: Mendelian randomization; MR-RAPS: Mendelian Randomization Robust Adjusted Profile Score; MR-PRESSO: Mendelian Randomization Pleiotropy Residual Sum and Outlier; SE: standard error; UL: upper limit; WM: weighted median.

**Exposure**		**Outcome**		**Selection of GVs**	**No. of GVs**	**MR method**	**Beta**				**Odds ratio**			**p-value**	**Cochran's Q**		**MR-Egger**			**Outlier detected**	**Sex difference**	
**Trait**	**Sex**	**Trait**	**Sex**				**Estimate**	**SE**	**95% CI LL**	**95% CI UL**	**Estimate**	**95% CI LL**	**95% CI UL**		**Statistic**	**p-value**	**Intercept**	**Intercept p-value**	**I**^**2**^_**GX**_	**by MR-PRESSO**	**z statistic**	**p-value**
Men																						
Basal metabolic rate	Men	Survival to 99th percentile of age	Overall	All	202	IVW (random)	−0.087	0.095	−0.275	0.100	0.916	0.760	1.105	0.359	259.518	0.003	–	–	–	–	0.420	0.674
				All	202	WM	−0.217	0.136	−0.484	0.050	0.805	0.617	1.051	0.111	–	–	–	–	–	–	1.098	0.272
				All	202	MR-Egger	−0.339	0.280	−0.888	0.210	0.713	0.412	1.234	0.226	258.342	0.003	0.008	0.340	0.838	–	0.010	0.992
				All	202	MR-RAPS	−0.089	0.085	−0.256	0.078	0.914	0.774	1.081	0.294	–	–	–	–	–	–	0.474	0.636
				All	201	MR-PRESSO (corrected)	−0.062	0.093	−0.245	0.122	0.940	0.783	1.130	0.510	–	–	–	–	–	rs768023	0.242	0.809
				Exclusion of potentially confounded GVs	186	IVW (random)	−0.042	0.102	−0.241	0.158	0.959	0.786	1.172	0.684	231.722	0.011	–	–	–	–	0.785	0.433
				Exclusion of potentially confounded GVs	186	WM	−0.185	0.145	−0.468	0.099	0.831	0.626	1.104	0.202	–	–	–	–	–	–	1.622	0.105
				Exclusion of potentially confounded GVs	186	MR-Egger	−0.126	0.315	−0.744	0.491	0.881	0.475	1.634	0.689	231.621	0.010	0.003	0.776	0.791	–	0.461	0.645
				Exclusion of potentially confounded GVs	186	MR-RAPS	−0.042	0.093	−0.224	0.139	0.958	0.799	1.149	0.646	–	–	–	–	–	–	0.864	0.388
				Exclusion of potentially confounded GVs	185	MR-PRESSO (corrected)	−0.011	0.099	−0.206	0.185	0.989	0.814	1.203	0.915	–	–	–	–	–	rs768023	0.755	0.450
Basal metabolic rate	Men	Survival to 90th percentile of age	Overall	All	204	IVW (random)	−0.098	0.063	−0.221	0.026	0.907	0.801	1.026	0.120	288.099	7.92E−05	–	–	–	–	0.381	0.704
				All	204	WM	−0.048	0.086	−0.217	0.122	0.953	0.805	1.129	0.581	-	-	-	-	-	-	0.071	0.943
				All	204	MR-Egger	−0.313	0.179	−0.665	0.039	0.731	0.514	1.040	0.081	285.783	9.46E−05	0.007	0.201	0.832	-	0.422	0.673
				All	204	MR-RAPS	−0.100	0.054	−0.205	0.005	0.905	0.814	1.005	0.061	–	–	–	–	–	–	0.442	0.659
				All	203	MR-PRESSO (corrected)	−0.082	0.062	−0.203	0.040	0.921	0.816	1.040	0.186	–	–	–	–	–	rs768023	0.622	0.534
				Exclusion of potentially confounded GVs	188	IVW (random)	−0.104	0.068	−0.236	0.029	0.902	0.789	1.030	0.126	262.112	0.0002	–	–	–	–	0.793	0.428
				Exclusion of potentially confounded GVs	188	WM	−0.010	0.092	−0.189	0.170	0.991	0.828	1.185	0.917	–	–	–	–	–	–	0.491	0.623
				Exclusion of potentially confounded GVs	188	MR-Egger	−0.305	0.201	−0.699	0.089	0.737	0.497	1.093	0.129	260.522	0.0003	0.006	0.287	0.783	–	1.035	0.301
				Exclusion of potentially confounded GVs	188	MR-RAPS	−0.106	0.058	−0.220	0.007	0.899	0.802	1.008	0.067	–	–	–	–	–	–	0.934	0.350
				Exclusion of potentially confounded GVs	187	MR-PRESSO (corrected)	−0.085	0.066	−0.215	0.046	0.919	0.806	1.047	0.201	–	–	–	–	–	rs768023	0.796	0.426
Women																						
Basal metabolic rate	Women	Survival to 99th percentile of age	Overall	All	202	IVW (random)	−0.029	0.100	−0.225	0.167	0.971	0.798	1.182	0.770	249.285	0.012	–	–	–	–		
				All	202	WM	−0.0003	0.143	−0.280	0.280	0.9997	0.756	1.322	0.998	–	–	–	–	–	–		
				All	202	MR-Egger	−0.335	0.293	−0.909	0.238	0.715	0.403	1.269	0.252	247.751	0.012	0.009	0.266	0.867	–		
				All	202	MR-RAPS	−0.030	0.091	−0.209	0.149	0.971	0.812	1.161	0.743	–	–	–	–	–	–		
				All	202	MR-PRESSO*	−0.029	0.100	−0.227	0.168	0.971	0.797	1.183	0.770	–	–	–	–	–	–		
				Exclusion of potentially confounded GVs	183	IVW (random)	0.074	0.107	−0.136	0.284	1.077	0.873	1.329	0.490	218.214	0.034	–	–	–	–		
				Exclusion of potentially confounded GVs	183	WM	0.157	0.153	−0.143	0.457	1.170	0.867	1.579	0.305	–	–	–	–	–	–		
				Exclusion of potentially confounded GVs	183	MR-Egger	0.087	0.338	−0.575	0.749	1.091	0.563	2.114	0.797	218.212	0.031	–0.0004	0.968	0.836	–		
				Exclusion of potentially confounded GVs	183	MR-RAPS	0.076	0.100	−0.120	0.271	1.079	0.887	1.311	0.447	–	–	–	–	–	–		
				Exclusion of potentially confounded GVs	182	MR-PRESSO (corrected)	0.098	0.105	−0.109	0.305	1.103	0.896	1.356	0.353	–	–	–	–	–	rs599004		
Basal metabolic rate	Women	Survival to 90th percentile of age	Overall	All	203	IVW (random)	−0.063	0.067	−0.194	0.067	0.939	0.824	1.070	0.343	277.242	0.0003	–	–	–	–		
				All	203	WM	−0.057	0.093	−0.239	0.126	0.945	0.787	1.134	0.542	–	–	–	–	–	–		
				All	203	MR-Egger	−0.202	0.193	−0.580	0.177	0.817	0.560	1.194	0.297	276.441	0.0003	0.004	0.446	0.851	–		
				All	203	MR-RAPS	−0.065	0.058	−0.178	0.048	0.937	0.837	1.050	0.262	–	–	–	–	–	–		
				All	201	MR-PRESSO (corrected)	−0.027	0.063	−0.152	0.098	0.974	0.859	1.103	0.673	–	–	–	–	–	rs3800228, rs599004		
				Exclusion of potentially confounded GVs	184	IVW (random)	−0.026	0.071	−0.164	0.113	0.974	0.848	1.119	0.714	237.752	0.004	–	–	–	–		
				Exclusion of potentially confounded GVs	184	WM	0.056	0.098	−0.136	0.248	1.058	0.873	1.281	0.568	–	–	–	–	–	–		
				Exclusion of potentially confounded GVs	184	MR-Egger	0.002	0.218	−0.426	0.430	1.002	0.653	1.537	0.994	237.728	0.003	–0.0008	0.894	0.812	–		
				Exclusion of potentially confounded GVs	184	MR-RAPS	−0.026	0.063	−0.150	0.097	0.974	0.861	1.102	0.674	–	–	–	–	–	–		
				Exclusion of potentially confounded GVs	183	MR-PRESSO (corrected)	−0.009	0.069	−0.145	0.126	0.991	0.865	1.135	0.895	–	–	–	–	–	rs599004		

## Discussion

Using MR and outcomes that obviate survival bias, we have shown that genetically higher BMR was associated with reduced parental attained age. Validation using survival to old age also showed directionally similar results. Our results are consistent with observational studies in younger individuals that reported a higher BMR being positively associated mortality^8,9^ and some theories of ageing^14^.

From the perspective of disease etiology, cancer and cardiovascular diseases are two leading causes of death worldwide^23,24^ and contribute substantially to years of life lost^24,25^. In an MR study, BMR was shown to increase the risk of cancer^26^, which supports our findings. Excessive harmful reactive oxygen species produced at a higher rate of metabolism that cannot be compensated by timely cell repair are thought to be the underlying mechanism^27^. The role of BMR in the development of cardiovascular diseases is not well studied. However, in observational studies BMR is reported to be positively associated with systolic blood pressure^28,29^, an established risk factor for cardiovascular diseases, although causality between BMR and systolic blood pressure could not be ascertained. Some anti-obesity drugs that act via raising BMR, e.g., thyroid hormone and melanocortin-4 receptor agonists, have been reported to cause cardiovascular side effects^30,31^. Postulated mechanisms linking higher BMR and systolic blood pressure include increased cardiac output^28^ and excessive production of reactive oxygen species^29^.

We found an inverse association of BMR with parental attained age in both men and women with a stronger association in women than men. Investigation of the downstream effects of BMR may gain more insight. A sex-specific effect may have clinical implications because muscle mass, a readily modifiable component that contributes substantially to BMR^32^, differs by sex^22^. At the same body mass index, men have more muscle mass and less fat mass than women. Whether the disadvantage at the starting line in men of having more muscle mass could be compensated by the weaker effect of BMR on lifespan compared to women throughout their life is unclear.

BMR is a possible target of intervention for lifespan in several ways. For lifestyle aspects, resistance training has been suggested as increasing BMR^33^, possibly through building muscle mass^34^. Caloric restriction reduces BMR^35^ and appears to promote longer lifespan^36^, as also in line with the viewpoint of tradeoff between growth, reproduction, and longevity in evolutionary biology^37,38^. In animal studies, it has recently been suggested that metabolism may interact with life history traits, specifically growth and reproduction, to act as a constraint on maximizing fitness, i.e., lifetime reproduction^39^. During these processes, a higher metabolic rate was observed to be associated with reduced longevity. Protein intake is reported to raise insulin-like growth factor 1^40^, a hormone responsible for growth, which also increases BMR^41^. For therapies that alter hormonal levels, exogenous testosterone has been shown to increase BMR, largely through building muscle mass^42^. Raising thyroid hormones also leads to an increase in BMR^43^. Climate change towards global warming may have an impact on BMR. Prolonged heat exposure may impair thermoregulation, a process to maintain normal core temperature of the body by increasing BMR at low ambient temperature and vice versa^44^, thus causing a rise in core temperature^45,46^. BMR increases with core temperature^47^, although protein damage can occur and eventually inhibits BMR^45^.

The validity of an MR study relies on a few key assumptions. First, it requires GVs to be strongly associated with the exposure. To assure this, we only selected GVs associated with BMR at genome-wide significance (p-value < 5 × 10^–8^). Also, all GVs predicting BMR had an F-statistic ≥ 30, which is greater than the rule-of-thumb value of 10 for a non-weak instrument^48^. The I^2^_GX_ value for MR-Egger was approximately 0.9, which also reflects an overall non-weak set of instruments^49^. Second, it requires GVs not to be associated with the confounders of the GV-outcome association. Although it was not possible for us to perform an exhaustive check on all potential confounders, we assessed the association of GVs with five key potential confounders related to lifestyle and socioeconomic position in the UK Biobank and excluded relevant GVs in sensitivity analysis. The results were largely consistent. Third, it requires GVs to affect the outcome only via the exposure of interest, which can be violated in different ways. A GV can exhibit horizontal pleiotropy, i.e., influencing multiple exposures, which in turn affects the outcome through different pathways. To address this issue, we conducted sensitivity analysis using different MR methods, including the weighted median, MR-Egger, and MR Pleiotropy RESidual Sum and Outlier (MR-PRESSO), which gave directionally similar results. MR-Egger intercept tests also did not suggest the presence of directional horizontal pleiotropy. Besides, selection bias in MR can distort associations if the GV (GV predicting BMR) or the exposure itself (BMR) affects survival, which precludes subjects from being recruited into the underlying genome-wide association studies (GWASs)^50^. This is an inherent problem of MR studies because of the time lag between randomization and recruitment into the study^21^. Selection bias in MR can also occur when a competing risk that shares common causes with the outcome of interest also affects survival^50^. However, our MR analysis benefits from using parental attained age as an outcome, which is not subject to such concerns.

This study has certain limitations. First, data on BMR was derived from impedance measurement using a body composition analyzer instead of being measured using the gold standard, indirect calorimetry. However, good estimation may be achieved by including body composition in the prediction formula^51^. Second, weak instrument bias may be present, which generally biases MR estimates towards the null in two-sample MR, provided that there is no sample overlap^52^. In our study, both the exposure and primary outcome used participants from the UK Biobank. However, the use of IVW and weighted median methods in two-sample MR using single sample are considered acceptable for large-sample studies^53^. MR Robust Adjusted Profile Score (MR-RAPS) complemented the results under possible weak instrument bias. In contrast, the MR-Egger estimate should be interpreted with caution in view of the insufficiently large I^2^_GX_ value^53^, with a maximum of 0.89 in the analysis of parental attained age. However, most MR-Egger estimates were directionally similar to the IVW one. Third, we assumed a linear association between BMR and parental attained age. With summary-level data only, we were unable to verify this assumption. Fourth, population stratification can introduce genetic confounding. However, the underlying GWASs we used restricted analysis only to individuals of European descent. Besides, they were also adjusted for principal components for ancestry. Fifth, the MR estimates in our study represent only the lifelong effect of BMR on parental attained age. Whether short-term interventions on BMR could produce similar effects requires further investigation. Sixth, our findings may not be generalized to populations other than those of European descent. However, causes should be consistent across populations though relevancy is an issue^54^, especially because body composition across population varies. Seventh, canalization refers to the developmental compensation arisen from the effect of the GV. However, its existence in our study is unknown. Further, it also biases MR estimates towards the null, which could not explain the observed associations. Eighth, sex-specific GWASs were available for the exposure and primary outcome but not the additional outcome.

In conclusion, our results showed that higher BMR might reduce lifespan in a sex-specific manner. This might have a practical implication on recommending the optimal level of physical activity, especially resistance training and muscle mass building as well as dietary patterns, hormonal treatments, and environmental factors that raise BMR. The underlying mechanism by which BMR is related to major causes of death is worth further investigation.

## Methods

We conducted an MR study of the association of BMR with parental attained age as the primary outcome using a two-sample MR approach by applying genetic predictors of the exposure to GWASs of outcomes with summary statistics. As an instrumental variable analysis, MR requires fulfillment of several important assumptions to give valid results. First, the GVs must be strongly associated with the exposure. Second, the GVs must not confound the GV-outcome association. Third, the GVs must be independent of the outcome given the exposure. In particular, given, we used two-sample methods applied in a one-sample study, albeit, in a large study, we checked for validity of the estimates^53^ and we additionally used survival to old age as an additional outcome, because the cases does not overlap with source study for the exposure.

### Data source

#### Basal metabolic rate

Genetic predictors of BMR were retrieved from UK Biobank summary statistics provided by Neale Lab^55^. The UK Biobank is a large-scale population-based prospective study, which aimed to enroll > 500,000 participants aged between 40 and 69 years from 2006 to 2010 in Great Britain^56^. BMR (in kilojoule) was estimated using a body composition analyzer (Tanita BC418MA Body Composition Analyzer) (https://biobank.ctsu.ox.ac.uk/crystal/refer.cgi?id=1421), which is based on Dual-energy X-ray absorptiometry using bioelectrical impedance analysis (https://www.tanita.com/es/.downloads/download/?file=855638086&fl=en_US). The analyzer provides BMR derived mainly from fat-free mass using a proprietary regression formula, which highly correlates to that measured using indirect calorimetry (r = 0.9, p < 0.0001) (https://www.tanita.com/es/.downloads/download/?file=855638086&fl=en_US). The Neale Lab obtained quality-controlled sex-specific genetic associations in unrelated participants with BMR using multivariable linear regression, adjusted for age, age x age, and the first 20 principal components in up to 361,194 individuals (54% women) of white British ancestry. The estimates were in standard deviations from rank-inverse-normalization of residuals of the regression (phenotype code, 23105_irnt).

#### Parental attained age

Genetic associations with parental (father’s and mother’s) attained age, where the age of parents (in years) was reported by their offspring, were retrieved from a GWAS of lifespan^57^. This GWAS analyzed attained age for 415,311 fathers and 412,937 mothers of participants of European descent from the UK Biobank^56^ after excluding those who reported themselves as adopted or had premature death of parents defined as a father who died before 46 years old and mother who died before 57 years old. The genetic associations were adjusted for age, sex, assessment centre, and array type. The estimates were expressed as rank-normalized Martingale residuals of the Cox’s proportional hazards regression, with a positive value representing a reduced attained age.

#### Survival to the 90th and 99th percentile of ages

Genetic associations with odds of survival to the 90th and 99th percentile of ages or above compared to the 60th percentile of age or below, where percentiles of age were country-, sex-, and birth cohort-specific, were retrieved from a meta-analysis of GWASs of survival to old age^58^. This study contains 11,262 cases of survival to the 90th percentile of age, 3,484 cases of survival to the 99th percentile of age, and 25,483 controls of survival to the 60th percentile of age in people of European descent. The genetic associations were adjusted for clinical site, known family relationships, and/or the first four principal components. The estimates were in log odds ratios of survival.

### Selection of genetic variants

We excluded GVs for BMR that are non-biallelic, rare (minor allele frequency < 0.01), or not in Hardy–Weinberg equilibrium (p-value for Chi-squared test < 0.05). Only GVs that predicted BMR at p-value < 5 × 10^–8^ were retained. We then obtained the uncorrelated GVs, i.e., GVs not in linkage disequilibrium, with r^2^ < 0.001 using the “clump_data” function in the “TwoSampleMR” R package^59^. GVs unavailable for the outcome were discarded.

### Assessment of confounding

We examined whether the GVs for BMR were associated with five key potential confounders of the GVs on outcome related to socioeconomic status and lifestyle attributes, including a measure of deprivation (Townsend index (phenotype code, 189)), age at completion of full-time education (phenotype code, 845), number of days per week walked for 10 min or more (phenotype code, 864), current smoking (phenotype code, 1239), and alcohol intake frequency (phenotype code, 1558) using summary statistics from the UK Biobank provided by the Neale Lab^55^, at Bonferroni-corrected significance, i.e., 0.05 / (number of GVs x number of traits).

### Statistical analysis

We harmonized the effect allele of a GV for exposure and outcome based on the allele letters in general. The Neale Lab reported the reference alleles on the forward strand^55^. Pilling et al. and Deelen et al. did not report the strand orientation but provided the effect allele frequency^57,58^. We therefore only kept palindromic GVs with effect allele frequency ≤ 0.45 and ≥ 0.55 (i.e., not close to 0.50) in the analysis.

GV-specific Wald estimates were calculated as the ratio of the effect size of the GV-outcome association to that of the GV-exposure association^60^. The corresponding standard error was approximated from the first term of Fieller’s theorem^61^.

We computed the F-statistic, which reflects the strength of genetic instrument, for each GV using an approximation, i.e., by dividing the squared effect size by the squared standard error of GV-exposure association^49^. An F-statistic of > 10 indicates that the genetic instrument is not weak.

In the primary analysis, we meta-analyzed GV-specific Wald estimates using IVW with multiplicative random effects, which assumed no measurement error for the exposure^49^ and balanced pleiotropy^62^, using the “MendelianRandomization” R package^63^. The Cochran’s Q statistic quantifies the hetereogeneity among the genetic instruments and serves as an indicator of potential horizontal pleiotropy^59,64^.

As a sensitivity analysis to address potential horizontal pleiotropy, where GVs predicting the exposure of interest affect the outcome via pathways other than the exposure of interest itself, we employed the weighted median and MR-Egger methods using the “MendelianRandomization” R package^63^, MR-PRESSO method using the “MRPRESSO” R package (with 10,000 simulations)^65^, and MR-RAPS method using the “mr.raps” R package^66^. The weighted median method provides a valid estimate when > 50% of weight is from valid GVs^67^. The MR-Egger method provides a valid estimate as long as the “instrument strength independent of direct effect”^49,68^ and “no measurement error for the exposure” assumptions hold^49^. A non-zero MR-Egger intercept indicates the presence of directional pleiotropy or violation of the “instrument strength independent of direct effect” assumption, which biases the IVW estimate^49^. The I^2^_GX_ value of MR-Egger reflects the overall strength of the genetic instrument set and a higher value close to unity is more desirable in order to minimize the regression dilution bias arisen from measurement error^49^. When conducting one-sample MR, estimates should be valid with the sample size here, as long as the I^2^_GX_ > 97% for MR-Egger^53^. The MR-PRESSO method adopts the IVW method but detects directional pleiotropy statistically and corrects it by removing outliers^65^. The MR-RAPS method assumes balanced pleiotropy and that the pleiotropic effects are normally distributed^66^. It also takes into account measurement error for the exposure that can lead to weak instrument bias.

We repeated all analyses by removing GVs associated with potential confounders at Bonferroni-corrected significance as additional sensitivity analysis.

To visualize the results for the primary outcome, we additionally presented the MR estimates by converting the rank-normalized Martingale residuals into years of life lost using an established approximation, i.e., multiply by 2.2869 and 2.5863 for fathers and mothers, respectively, to account for the genetic associations given offspring only share half their genetic endowment with each parent, then multiply by 10 in accordance with a long-standing actuarial rule of thumb recently verified^69^. To obtain the overall associations, we also meta-analyzed the sex-specific estimates of years of life lost.

Any sex difference in years of life lost was assessed using a z-test^70^.

Post hoc power calculations were performed using an online calculator^71^, which is based on the approximation that the sample size for an MR study is the sample size for exposure on outcome divided by the proportion of variance in exposure explained by the GVs^72^. The proportion of variance in BMR explained by each GV was estimated by an approximation as (effect size of the genetic associations with BMR)^2^ × 2 x minor allele frequency x (1 – minor allele frequency)^73^. All GV-specific proportions of variance were summated to give the overall one.

All statistical analyses were conducted using R (Version 4.0.3, The R Foundation for Statistical Computing Platform, Vienna, Austria).

### Eithics approval and consent to participate

We only used publicly available summary-level data in the current study. No original data was collected. Ethical approval including written informed consent from individual participants can be found in the original publications.

## Supplementary Information

Supplementary Tables.

## Data Availability

The datasets generated during and/or analyzed during the current study are available from the website of the Neale Lab (http://www.nealelab.is/uk-biobank/), a GWAS of parental attained age (https://figshare.com/articles/dataset/Plling_et_al_2017_UKB_parents_attained_age_GWAS/5439382/2), and a GWAS of survival to old age (https://www.ebi.ac.uk/gwas/publications/31413261).

## Abbreviations

BMR: Basal metabolic rate
CI: Confidence interval
GV: Genetic variant
GWAS: Genome-wide association study
IVW: Inverse-variance weighting
MR: Mendelian randomization
MR-RAPS: Mendelian randomization robust adjusted profile score
MR-PRESSO: Mendelian randomization pleiotropy residual sum and outlier
